# Acute double coronary occlusion and its misleading presentation: An unusual case report

**DOI:** 10.1016/j.amsu.2021.103133

**Published:** 2021-12-03

**Authors:** Maryem Assamti, Ilham Benahmed, Nabila Ismaili, Noha El Ouafi

**Affiliations:** a Department of Cardiology, Mohamed VI University Hospital, Faculty of Medicine and Pharmacy of Oujda, Mohamed First University, Oujda, Morocco; b Laboratory of Epidemiology and Clinical Research, Mohamed First University, Oujda, Morocco

**Keywords:** acute Coronary syndrome, Coronary atherosclerosis, Multivessel coronary artery disease

## Abstract

Acute simultaneous double coronary occlusion is an extremely rare condition with an unspecific presentation.

We report a case of a 57-year-old male, with undiagnosed diabetes mellitus, presenting with acute epigastralgia and vomiting associated with dynamic electrocardiographic changes. He was hemodynamically stable. Emergency coronary angiogram showed a total occlusion of both proximal left circumflex and mid left anterior descending coronary artery. Since the EKG indicated minimal ST-segment elevation in the lateral leads as well as an ST depression in the inferior leads, we performed a percutaneous coronary intervention of both the LCx and LAD, using a floppy guidewire.

Similar cases of multiple simultaneous coronary occlusions are reported in literature, yet the accurate incidence and physiopathology of this occurrence is still uncertain.

Although this condition is associated with serious complications, our case evolved favorably due to prompt management.

## Highlights

• Acute coronary syndromes are a major cause of cardiovascular mortality.
• Simultaneous acute double coronary occlusion is a rare condition.
• Its presentation is often misleading.
• Complications are frequent and fatal.
• Mortality may be avoided by an early diagnosis and invasive management.

## 1 Introduction

Despite all the improvement of therapeutic and preventive measures, acute myocardial infarction, either with or without ST segment elevation, remains the first cause of death worldwide.

STEMI is by definition related to an acute occlusion of an epicardial coronary artery, leading to a localized myocardial ischemia and necrosis. The main pathologic finding is atherothrombotic obstruction of a coronary artery [1].

Acute multicoronary occlusion (AMCO) is an extremely rare situation characterized by a severe clinical presentation, and an unpredictable outcome due to the impairment of an important area of myocardium.

The electrocardiogram is the main primary tool to identify the culprit artery in acute coronary syndromes. However, in case of simultaneous double coronary occlusion, the electric findings are extremely heterogeneous and may be attenuated [2].

Our case has been reported in line with THE SCARE 2020 criteria [3].

## 2 Case presentation

**Fig. 1:**
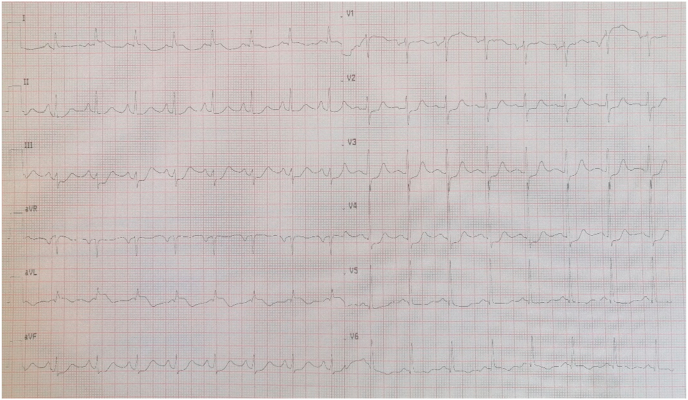
EKG showing an ST segment elevation in lateral leads, and an ST depression in apical and inferior leads.

**Fig. 2:**
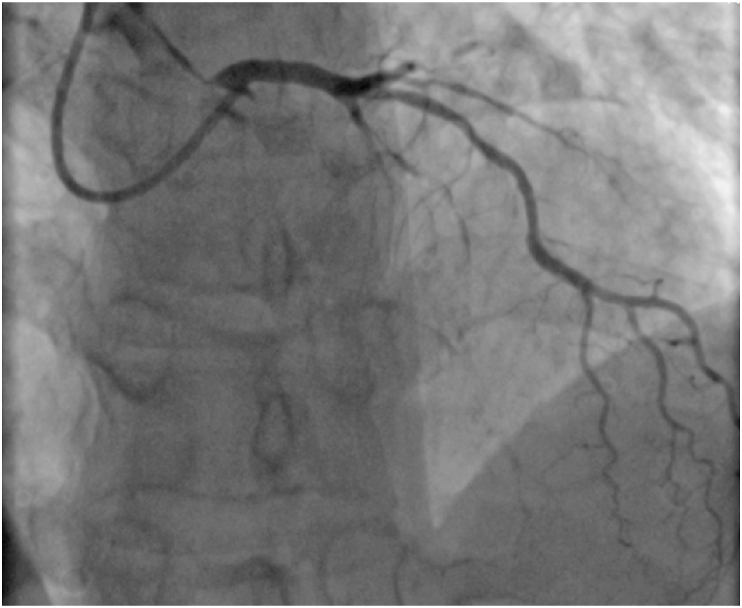
Coronary angiogramm showing a total occlusion of mid left anterior descending coronary artery and an occlusion of the proximal left circumflex.

**Fig. 3:**
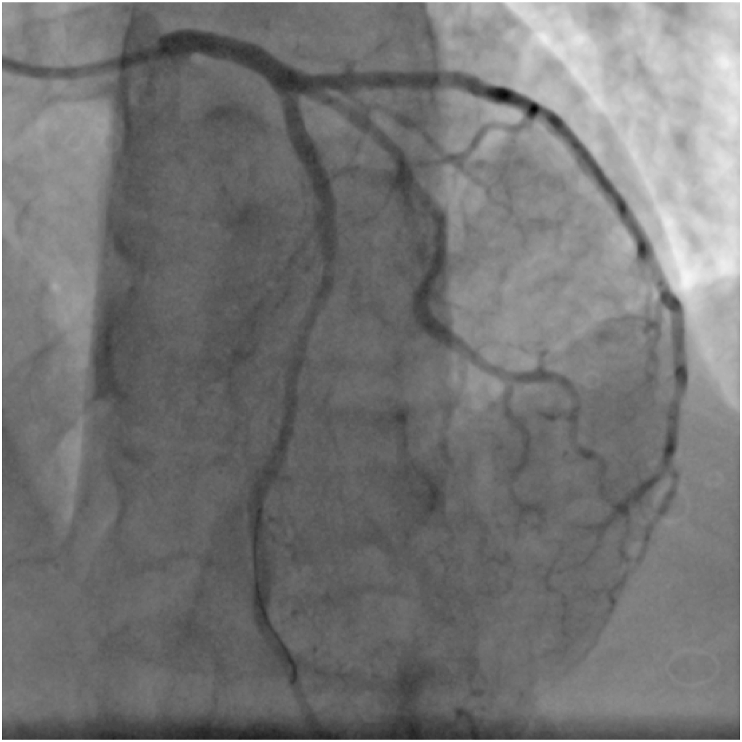
Coronary angiogramm showing a successful PCI of the LAD artery and the circumflex artery, after predilation and deployment of drug eluting stents (DES).

A 57-years-old man with a history of smoking interrupted 6 months prior, and no other significant past medical history, was admitted in the emergency department for acute epigastralgia and vomiting. On arrival, he was alert and oriented with a blood pressure of 130/90 mmHg, a heart rate of 85/minute, and a respiratory rate of 19 breaths/minute. Cardiovascular examination was unremarkable. His blood glucose was 260 mg/dl without acetonuria. His electrocardiogram revealed normal sinus rhythm with ST segment depression in lateral, inferior, and posterior leads. His cardiac enzymes were elevated (First Troponin I at 755 ng/L, the second at 1600 ng/l), serum creatinine and electrolyte levels were normal. Echocardiography demonstrated an akinesia of the lateral wall with a left ventricular ejection fraction of 50%. The patient was admitted to the intensive care unit and prepared for cardiac catheterization. He received 300 mg of aspirin, 600 mg of clopidogrel and full dose 6000 UI of enoxaparine subcutaneously, with a slight regression of the pain. An electrocardiogram performed 6 h after his admission showed an ST elevation in the lateral and posterior leads (Fig. 1). Urgent coronary angiography revealed a concomitant occlusion of the mid LAD and the proximal Left circumflex artery with a collateral flow from the RCA (Fig. 2). We proceeded with primary per cutaneous intervention of the circumflex occlusion that was crossed by a floppy guidewire and predilated with a 2 × 15 mm balloon, then we deployed a 2,75 × 38 mm drug eluting stent (DES). Afterward, we proceeded to the PCI of the LAD using the same procedure to deploy a 2,75/28 mm DES. The angiographic result showed Thrombolysis In Myocardial Infarction III flow in both arteries (Fig. 3). Unfortunately, IVUS was not available. Aside from an inaugural diabetes mellitus (an incidental finding during this case presentation), there was no clinical or biological feature suggestive of an underlining condition predisposing to multiple thrombosis. The clinical course was uneventful and the patient was discharged well on day 4.

## 3 Discussion

STEMI with simultaneous double or multivessel occlusion is an uncommon and potentially lethal clinical condition associated with unspecific electrocardiographic (ECG) patterns. That may occur in about 2.5% of patients with ST-segment elevation myocardial infarction (STEMI) [4].

The mechanisms of multiple coronary occlusion is controversial. Simultaneous rupture of different coronary plaque is a possible mechanism, that may be precipitated by the prothrombotic and inflammatory pool associated with acute myocardial infarction. Local hemodynamic changes related to acute systolic dysfunction of atrioventricular asynchronism may also be a risk factor [5].

Multiple coronary thrombosis may be encountered in cases of constitutional or acquired thrombophilia such as in essential thrombocythemia [6], polycythemia vera [7] or hyperhomocysteinemia [8]. Other identifiable predisposing factors include coronary ectasia [9], vasospasm [10]and cocaine use [11]. Therefore, it is reasonable to investigate an underlining disease in these cases.

The 12-lead ECG is of importance for determining the culprit coronary artery and for localizing the myocardial infarction area. However, in case of multivessel coronary thrombosis electrocardiographic findings may be misleading. Inferior and/or anterior ST elevation may be observed in about 30% of cases, while an isolated lateral ST elevation is noted in only 2% of cases [4].

M. Vives-Borrá and al conducted an interesting experimental study on swine. They induced simultaneous double vessel occlusion in the main three coronary arteries, and analyzed the different electrocardiographic patterns. This study showed a summation effect of the QRS widening and ST segment depression in concomitant infero-lateral (RC + LCx) ischemia. However, LAD involvement induced a decrease in ST segment elevation, particularly in V3 and V5 leads [2], as illustrated by our case too. These summation or cancellation effects are related, respectively, to the alignment or the contraposition of the underlining ischemic areas [2].

According to recent systematic review, these patients often present with cardiogenic shock in about 36–41% of cases, they are also at high risk of ventricular arrhythmia in 23–25% of cases, and bradyarrhythmia in about 20% of cases [4,12].These threatening complications require prompt management.

The early diagnosis in our case, and urgent revascularization prevented the deterioration of the patient's clinical course. This underscores the attitude of early reperfusion therapy (first 2 h) recommended by the latest NSTEMI guidelines, in case of very high risk patients [13]. Since the catheterization lab is not permanently available in our context, a close electrocardiogram monitoring is crucial regardless of the clinical symptoms.

The reported mortality for simultaneous multi-vessel coronary thrombosis is low and varies from 2 to 5% [3,11],which contrasts with the severity of this condition. The most rational explanation is that most patients present with initial cardiac arrest. Autopsic findings confirm this hypothesis, since multiple coronary artery thrombosis was found in approximately 50% of out of hospital cardiac arrest patients [1].

## 4 Conclusion

This unusual case demonstrates that ECG findings do not always support angiographic findings. During coronary angiography, identifying a culprit lesion may be delicate and cardiologists should be prepared to manage multiple coronary occlusions.

## Provenance and peer review

Not commissioned, externally peer-reviewed.

## Annals of medicine and surgery

The following information is required for submission. Please note that failure to respond to these questions/statements will mean your submission will be returned. If you have nothing to declare in any of these categories then this should be stated.

### Please state any sources of funding for your research

No funding to disclose.

### Ethical approval

No identifying data was published.

### Author contribution

ASSAMTI M.: wrote the manuscript.

BENAHMED I.: revised the manuscript.

ISMAILI N.: revised the manuscript.

EL OUAFI N.: performed the procedure and wrote the manuscript.

### Consent

Written informed consent was obtained from the patient for publication of this case report and accompanying images. A copy of the written consent is available for review by the Editor-in-Chief of this journal on request.

### Registration of research studies

1. Name of the registry:
2. Unique Identifying number or registration ID:
3. Hyperlink to your specific registration (must be publicly accessible and will be checked)

### Guarantor

ASSAMTI Maryem.

## Declaration of competing interest

The authors state that they have no conflicts of interest for this report.
